# Interaction of EGFR to δ-catenin leads to δ-catenin phosphorylation and enhances EGFR signaling

**DOI:** 10.1038/srep21207

**Published:** 2016-02-17

**Authors:** Yongfeng He, Taeyong Ryu, Nensi Shrestha, Tingting Yuan, Hangun Kim, Hridaya Shrestha, Young-Chang Cho, Young-Woo Seo, Woo Keun Song, Kwonseop Kim

**Affiliations:** 1 College of Pharmacy and Research Institute for Drug Development, Chonnam National University, Gwangju 500-757, Korea; 2 College of Pharmacy and Research Institute of Life and Pharmaceutical Sciences, Sunchon National University, Sunchon 540-742, Korea; 3 College of Pharmacy, Chung-Ang University, Seoul 156-756, Korea; 4 Korea Basic Science Institute, Gwangju Center, Gwangju 500-757, Korea; 5 Department of Life Science, Bio Imaging and Cell Dynamics Research Center, Gwangju Institute of Science and Technology, Gwangju, Korea

## Abstract

Expression of δ-catenin reportedly increases during late stage prostate cancer. Furthermore, it has been demonstrated that expression of EGFR is enhanced in hormone refractory prostate cancer. In this study, we investigated the possible correlation between EGFR and δ-catenin in prostate cancer cells. We found that EGFR interacted with δ-catenin and the interaction decreased in the presence of EGF. We also demonstrated that, on one hand, EGFR phosphorylated δ-catenin in a Src independent manner in the presence of EGF and on the other hand, δ-catenin enhanced protein stability of EGFR and strengthened the EGFR/Erk1/2 signaling pathway. Our findings added a new perspective to the interaction of EGFR to the E-cadherin complex. They also provided novel insights to the roles of δ-catenin in prostate cancer cells.

Epidermal growth factor receptor (EGFR) is a member of the ErbB family of receptors, which consists of 4 closely related receptor tyrosine kinases: EGFR (ErbB-1), HER2/c-neu (ErbB-2), HER3 (ErbB-3) and HER4 (ErbB-4)^1^. Overexpression of EGFR is correlated with progression of many human cancers, including hormone refractory prostate cancer^2^,^3^,^4^. It is very important to investigate how EGFR is regulated in tumor cells, since it has an important role in tumorigenesis. While EGFR is up-regulated by Fbw-7 (F-box and WD repeat domain-containing 7), an ubiquitin ligase, and hypoxic condition^5^,^6^, it is down-regulated through various mechanisms, among which clathrin-dependent endocytosis, presenilin-1 and caspase-3 regulation are well studied^7^,^8^,^9^,^10^.

δ-Catenin belongs to the p120 catenin (p120ctn) subfamily of armadillo proteins, which is implicated in cell-cell adhesion and signal transduction. While p120ctn was originally identified as a major substrate for tyrosine phosphorylation^11^, δ-catenin was first identified as a binding partner for presenilin-1^12^. Despite their unrelated discoveries, they share similar structure and function, such as binding to juxta-membranous region of E-cadherin^13^,^14^. It has been reported that they competitively bind to E-cadherin in colorectal cancer cells^15^. It has also been demonstrated that δ-catenin was enhanced at both the mRNA and protein level and correlated with high Gleason scores, whereas protein expression of p120ctn was dramatically decreased along with increased Gleason scores in prostate cancer^16^,^17^. Loss of p120ctn was also observed in invasive breast cancer, which augmented EGFR signaling^18^. Contrarily, EGF-EGFR was reported to mainly phosphorylate p120ctn on its Y228 residue in a Src independent manner. However, this phosphorylation event was dispensable to junction formation^19^.

We currently investigated the relationship between δ-catenin and EGFR in order to delineate the potential connection between the enhanced EGFR expression in hormone refractory prostate cancer and the reciprocity of increased δ-catenin and decreased p120ctn expression during late stage prostate cancer. We found that the δ-catenin bound to EGFR in an EGF dependent manner. We demonstrated that δ-catenin was phosphorylated by EGF in an EGFR dependent, but Src independent manner. Our data indicated that δ-catenin stabilized EGFR protein expression and enhanced the EGFR/Ek1/2 signaling pathway.

## Results

### δ-Catenin-EGFR interaction was decreased by EGF treatment

**Figure 1:**
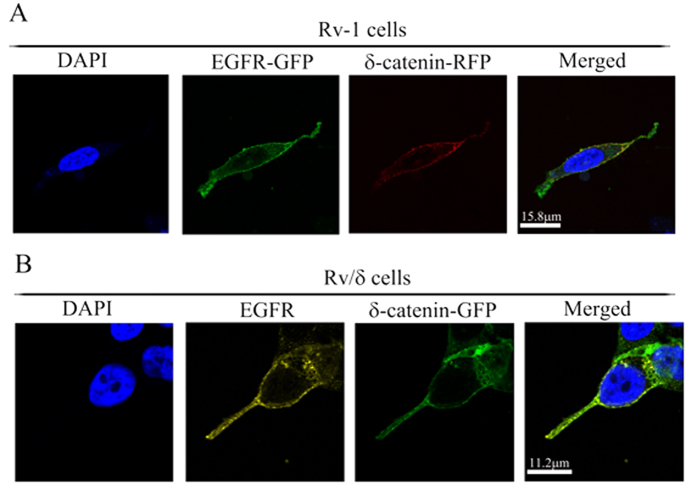
δ-Catenin was co-immunostained with EGFR in CWR22Rv-1 cells. (**A**) CWR22Rv-1 (Rv-1) cells were transfected with δ-Catenin-RFP and EGFR-GFP. The transfected cells were fixed with 4% PFA and subjected to confocal microscopy analysis. Red color stands for delta-catenin; Green color stands for EGFR. (**B**) Rv/δ cells were immunostained with EGFR antibody and fixed with 4% PFA followed by being subjected to confocal microscopy analysis. Green color represents delta-catenin; Yellow color represents EGFR. These staining were performed 3 times.

**Figure 2:**
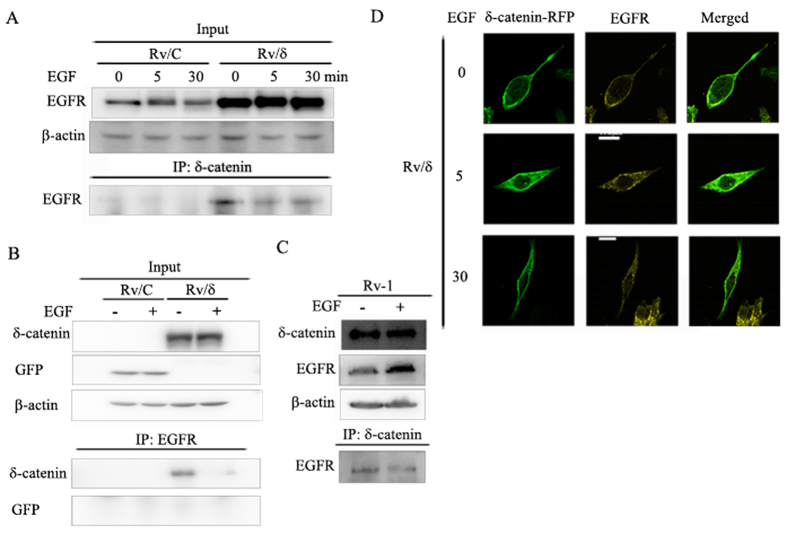
δ-Catenin interacted with EGFR in an EGF dependent manner in Rv/δ cells. (**A**) Rv/C and Rv/δ cells were treated with 100 nM EGF for different durations, as indicated. The cells were harvested and subjected to immunoprecipitation with anti-δ-catenin antibody followed by immunblotting with anti-EGFR antibody. (**B**) Reverse immunoprecipitation were performed with anti-EGFR antibody followed by anti-δ-catenin antibody. (**C**) Immunoprecipitation were performed using protein lysates from CWR22Rv-1 (Rv-1) cell line with anti-δ-catenin antibody followed by anti-EGFR antibody (**D**) Rv/δ cells were fixed with 4% PFA after being treated with EGF for different durations, as indicated. The fixed cells were immunostained with anti-EGFR antibody and subjected to confocal microscopy analysis. Green stands for δ-catenin; Yellow stands for EGFR. These experiments were performed 3 times.

We overexpressed δ-catenin-RFP and EGFR-GFP in CWR22Rv-1 cells in order to investigate the relationship between EGFR and δ-catenin. Interestingly, we observed co-localization of the two proteins (Fig. 1A). Additionally, we immunostained the Rv/δ cell line, a cell line stably expresses δ-catenin-GFP, with the anti-EGFR antibody. Co-localization of endogenous EGFR and δ-catenin-GFP was observed (Fig. 1B). To further confirm this data, we performed immunoprecipitation with the anti-δ-catenin antibody. We found that EGFR was detected in the purified δ-catenin immune-complex and interestingly, the interaction was reduced in response to EGF treatment (Fig. 2A). Reverse IP was conducted with the EGFR antibody. δ-Catenin was detected in the immune-complex as well (Fig. 2B). We additionally confirmed the interaction in Bosc23 and CWR22Rv-1 cell lines (Fig. S1). We also checked the interaction between endogenous δ-catenin and EGFR in CWR22Rv-1 cell line, the data was consistent with the ones from overexpression of δ-catenin and EGFR (Fig. 2C). Collectively, the data indicated that δ-catenin interacted with EGFR. The mechanism of EGF-induced reduction of the δ-catenin-EGFR interaction was evaluated by immunostaining EGF treated and untreated Rv/δ cells. As shown in Fig. 2D, EGF induced significant endocytosis of EGFR but did not dramatically affect the localization of δ-catenin. We confirmed this result by overexpressing δ-catenin-RFP and EGFR-GFP in Bosc23 cells (Fig. S1). Subsequent confocal microscopy revealed the same pattern.

### δ-Catenin was phosphorylated by EGF in an EGFR dependent but Src independent manner

**Figure 3:**
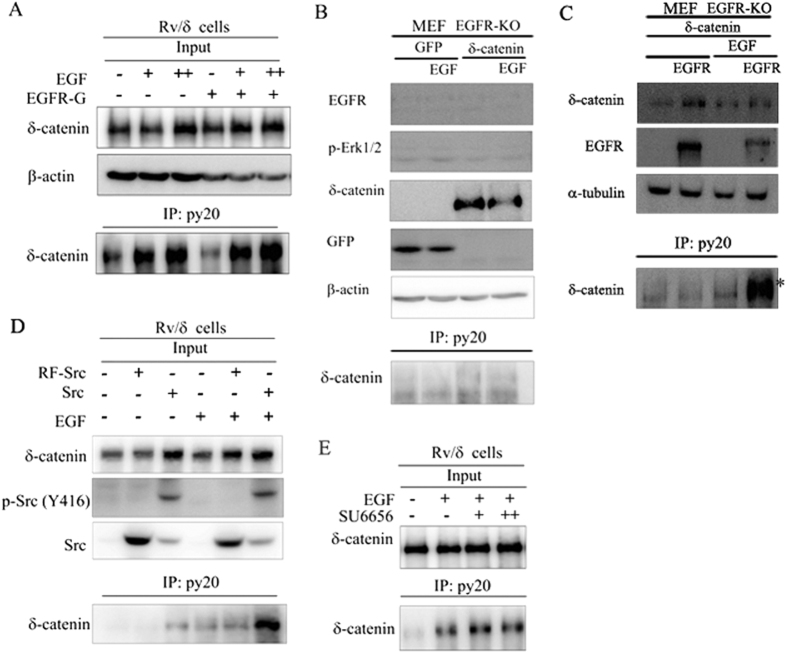
δ-Catenin could be phosphorylated by EGF in an EGFR dependent but Src independent manner. (**A**) δ-Catenin can be phosphorylated by EGF in a dosage dependent manner. Rv/δ cells were treated with different dosages of EGF after being cultured in serum free condition for 16 h. Cell lysates from both non-transfected and transfected Rv/δ cells were subjected to immunoprecipitation with anti-py20 antibody followed by immunoblotting with anti-δ-catenin antibody. (**B,C**) δ-Catenin could be phosphorylated by EGF in an EGFR dependent manner. MEF (EGFR Knockout) cell line was transfected with both GFP and δ-catenin-GFP. Transfected cells were treated with EGF and harvested to perform immunoprecipitation with anti-py20 antibody followed by δ-catenin antibody (**B**). MEF (EGFR Knockout) cell line was transfected with both EGFR and δ-catenin-GFP. Transfected cells were treated with EGF and harvested to perform immunoprecipitation with anti-py20 antibody followed by δ-catenin antibody (**C**). (**D,E**) δ-Catenin could be phosphorylated by EGF in an Src independent manner. Rv/δ cells were tranfected with either RF-Src (inactive form) or Src. The cells were treated with either serum free medium or EGF 100 nM for 5 minutes and harvested after the treatment. The lysates were subjected to immunoprecipitation with anti-py20 antibody followed by immunoblotting with anti-δ-catenin antibody (**D**). Rv/δ cells were treated with different dosages of SU6656, a Src kinase inhibitor, for 24 h prior to the EGF treatment. The cells were then harvested and subjected to immunoprecipitation with anti-py20 antibody followed by immunoblotting with anti-δ-catenin antibody (**E**). These experiments were performed more than three times.

The functional significance of the δ-catenin-EGFR interaction was firstly investigated by studying the effect of EGFR on δ-catenin. Src-EGFR synergism is a potential mechanism that contributes to an aggressive tumor phenotype^20^,^21^. Previously, we reported that Src kinases phosphorylated δ-catenin and affected its biological function^22^. Here, we hypothesized that EGF initiated tyrosine phosphorylation of δ-catenin. We accordingly performed immunoprecipitation with lysates from the Rv/δ cell line. As expected, δ-catenin was strongly phosphorylated in a dose dependent manner in response to EGF (Fig. 3A). However, overexpression of exogenous EGFR failed to significantly enhance the phosphorylation level, indicating that endogenous EGFR was sufficient for EGF-induced phosphorylation of δ-catenin. We next determined whether EGFR was required for EGF-induced phosphorylation of δ-catenin by overexpressing δ-catenin-GFP in EGFR-deficient MEF cells (EGFR Knockout) and immunoprecipitating the cell lysates. Tyrosine phosphorylated δ-catenin could not be observed. However, when we overexpressed EGFR in EGFR-deficient MEF cells, tyrosine phosphorylation of δ-catenin was detectable, suggesting that EGF-induced phosphorylation of δ-catenin was indeed EGFR-dependent (Fig. 3B,C). The role of Src kinase on EGF-induced δ-catenin phosphorylation was examined in Rv/δ cells with overexpressed RF-Src, a Src plasmid lacking kinase activity. Although the phosphorylation of δ-catenin was weaker than that in cells overexpressing Src, it showed no difference compared to non-transfected Rv/δ cells, indicating that the EGF-induced phosphorylation of δ-catenin was Src independent but that Src and EGFR had a synergic effect on phosphorylation of δ-catenin (Fig. 3D). We treated Rv/δ cells with SU6656, a Src kinase inhibitor to confirm this observation. Phosphorylated δ-catenin was expectedly observed in response to the treatment (Fig. 3E), suggestive that EGF-mediated tyrosine phosphorylation of δ-catenin was Src independent. Taken together, these data indicated that EGF-induced phosphorylation of δ-catenin required the involvement of EGFR but not Src.

### EGF failed to affect localization of δ-catenin and its interaction to E-cadherin

**Figure 4:**
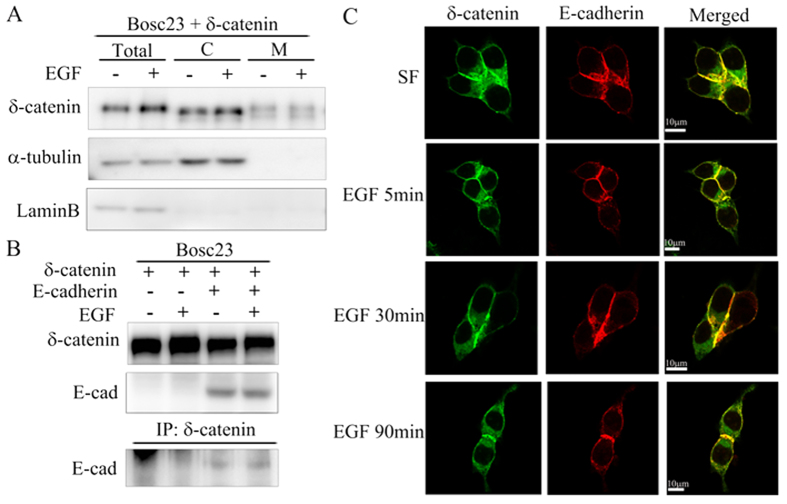
Tyrosine phosphorylation of δ-catenin failed to affect either its localization or its binding affinity to E-cadherin. (**A**) Bosc23 cells were transfected with δ-catenin-RFP. The transfected cells were treated with either serum free condition or EGF 100 nM for 5 minutes. Then, cells were harvested and subjected to performing fractionation experiments. α-Tubulin bands were from same lysate loaded on different gel. (**B**) Bosc23 cells were either transfected with δ-catenin-RFP or δ-catenin-RGF together with E-cadherin. The cells were then treated with EGF for 5 minutes and harvested to perform immunoprecipitation with anti-δ-catenin antibody followed by immunoblotting with E-cadherin antibody. (**C**) Rv/δ cells were seeded on coverslips in a 12 wells-plate. The cells were treated with 100 nM EGF for different durations, as indicated. Then cells were fixed by 4% PFA, immunostained with anti-E-cadherin antibody and subjected to confocal microscopy analysis. Green stands for δ-catenin; Red stands for E-cadherin. The western blots were performed more than three times whereas the immunostaining was performed more than five times.

We performed fractionation experiments in Bosc23 cells to detect whether phosphorylation of δ-catenin affected its localization. As shown in Fig. 4A, EGF treatment did not affect the localization of δ-catenin. We also examined the binding between δ-catenin and E-cadherin. However, we did not observe any dramatic changes in the presence of EGF (Fig. 4B). Moreover, on immunostaining Rv/δ cells with the anti-E-cadherin antibody, we observed strong co-localization of δ-catenin and E-cadherin at the cell-cell interface. EGF treatment for different time durations, as indicated, did not cause significant changes (Fig. 4C). Taken together, phosphorylation of δ-catenin induced by EGF affected neither δ-catenin localization nor its binding affinity to E-cadherin.

### δ-Catenin increased protein stability of EGFR in CWR22Rv-1 cells

**Figure 5:**
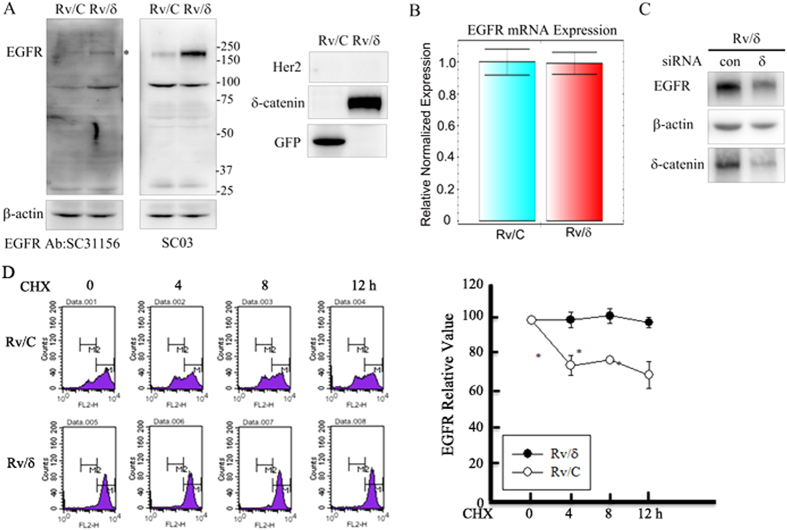
δ-Catenin enhanced EGFR protein stability. (**A**) EGFR protein expression was higher in Rv/δ than that in Rv/C cells. Lysates from both Rv/C and Rv/δ cells were subjected to immunoblotting with different anti-EGFR and β-actin antibodies. The experiment was performed five times (**B**) EGFR mRNA expression was not affected by overexpression of δ-catenin. mRNA were extracted from both Rv/C and Rv/δ cells were subjected to Q-PCR. The experiment was performed 5 times. (**C**) Knock down of delta-catenin decreased EGFR protein expression in Rv/δ cells. Either siRNA against δ-catenin or control siRNA were transfected into Rv/δ cells. After 72 h incubation, cells were harvested and subjected to immunoblotting with anti-EGFR, anti-δ-catenin and anti-β-actin antibodies. The experiment was performed three times. (**D**) Rv/C and Rv/δ cells were plated at 60 mm dish (1 × 10^6 cells/dish). After overnight incubation, 40 μ M of CHX was treated to each plate for indicated times (0, 4, 8, 12 h). The samples are applied to FACSCalibur™ for detection of the EGFR expression on the surface of each cell. Experiments were done for three times with independent trials. Left panel shows representative data of three independent experiments, and right panel reveals EGFR relative values from three independent experiments that were calculated by comparing with CHX-untreated group of each cell. *p < 0.05 (student t-test).

Although we failed to observe a dramatic effect of EGF/EGFR phosphorylation on δ-catenin (Localization, Binding affinity to E-cadherin), we observed a significant effect of δ-catenin on EGFR protein expression. We compared EGFR protein expression between Rv/δ cells and Rv/C control cells (stably expressing GFP) using 2 different EGFR antibodies. Surprisingly, EGFR protein level was much higher in Rv/δ cells than in Rv/C cells (Fig. 5A). We compared EGFR mRNA expression in the 2 cell lines by q-PCR to distinguish if increased EGFR protein expression by δ-catenin was a transcriptional or posttranslational event. There were no significant differences between the mRNA expression levels of the 2 cell lines (Fig. 5B), indicating that δ-catenin increased EGFR protein expression in a posttranslational manner. To confirm the role of δ-catenin on increased EGFR protein expression, we introduced specific siRNA targeting δ-catenin into Rv/δ cells. As shown in Fig. 5C, knockdown of δ-catenin decreased EGFR protein expression, confirming the role of δ-catenin in enhancing EGFR protein expression. We treated both Rv/C and Rv/δ cells with cycloheximide for different time durations to determine whether δ-catenin affected the stability of membrane EGFR protein by flow cytometry. As shown in Fig. 5D, membranous EGFR expression is more stabilized in Rv/δ cells than that in Rv/C cells, indicating that δ-catenin enhanced stability of EGFR in CWR22Rv-1 cells.

### δ-Catenin failed to block the EGFR endocytosis induced by EGF but affected the EGFR expression pattern in CWR22Rv-1 cell membrane

**Figure 6:**
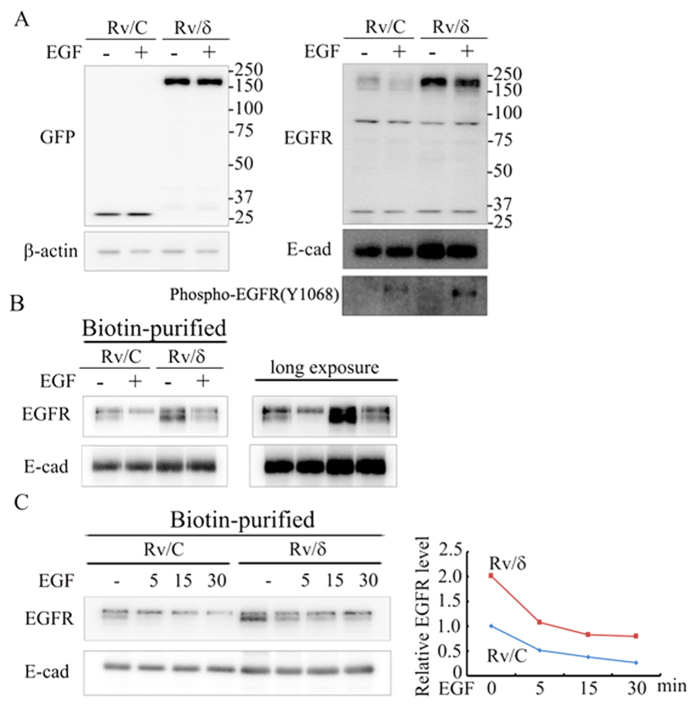
Membranous EGFR expression was higher in Rv/δ cells than that in Rv/C cells either in the presence of or absence of EGF treatment. (**A**) Rv/C and Rv/δ cells were cultured in serum free condition for 16 h followed by treatment of 100 nM EGF for 30 min. After the treatment, cells were collected and co-incubated with biotin for an hour. Then, the lysates were subjected to either immunoblotting (**A**) or biotinylation (**B**). (**B**) Membranous EGFR were detected by immunobloting with lysates purified by biotin and streptavidin. E-cadherin was considered as a loading control. (**C**) Rv/C and Rv/δ cells were cultured in serum free condition for 16 h followed by treatment of 100 nM EGF for different duration, as indicated. Lysate from these cells were subjected to biotinylation followed by immunoprecipitation with anti-EGFR and anti-E-cadherin antibodies. The density of the bottom EGFR band was measured and the pattern was demonstrated in the right panel. The experiment was performed three times.

EGFR can be regulated by different mechanisms, among which clathrin-dependent endocytosis has been well studied. We tested whether overexpression of δ-catenin could block the endocytosis of EGFR, in order to investigate how δ-catenin affected EGFR stability. Total EGFR and membranous EGFR levels were much higher in Rv/δ cells than that in Rv/C cells, in the presence of EGF (Fig. 6A,B), indicating that δ-catenin enhanced EGFR protein expression even in the presence of EGF. A time-course study of EGF treatment was done in the 2 cell lines to investigate the effects on the expression pattern (Fig. 6C). As we expected, the pattern of EGFR expression was consistent with what we observed in panel B. These results suggested that δ-catenin had a protective role on both total and membranous EGFR. Although membranous EGFR proteins in Rv/δ cells was significantly higher than that in Rv/C cells, the EGF-induced decreasing rate of membranous EGFR was similar in both cell lines (Fig. 6C, right panel). This indicated that δ-catenin failed to block the endocytosis of EGFR induced by EGF. It was notable that we detected a doublet of EGFR in the purified membrane lysates. Moreover, the expression patterns of the 2 bands were different between Rv/δ and Rv/C cells. As shown in Fig. 6B, in the absence of EGF, while expression of the upper EGFR band was higher than the lower band in Rv/C cells, the lower band was much higher than the upper band in Rv/δ cells. Furthermore, whereas the upper bands slightly changed in the presence of EGF, the lower bands were dramatically decreased in both cell lines, suggesting that the lower bands were more sensitive to EGF treatment. Taken together, these results suggested that while δ-catenin did not abolish clathrin-dependent endocytosis of EGFR, δ-catenin changed the pattern of membranous EGFR expression in Rv/δ cells.

### δ-Catenin may stabilize EGFR directly or through unknown factors

**Figure 7:**
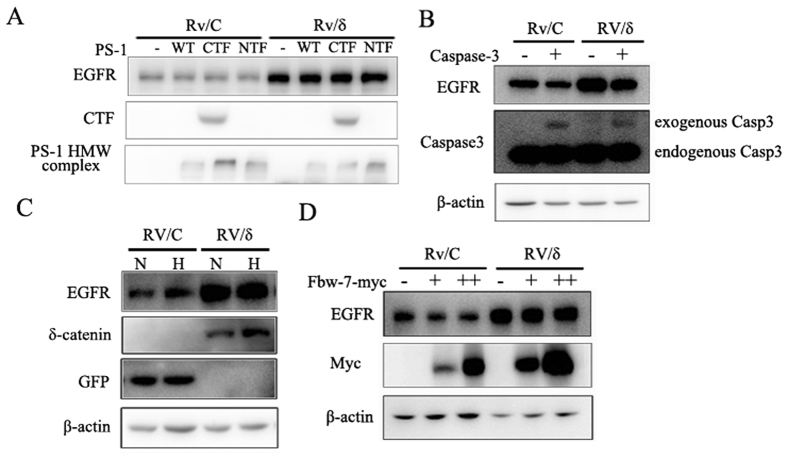
δ-Catenin neither protected EGFR degradation from caspase-3 or PS-1 nor enhance EGFR expression via hypoxic condition or Fbw-7. (**A**) δ-Catenin failed to protect EGFR from degradation mediated by PS-1. PS-1-GFP, its C-terminus or N-terminus construct was transfected into both Rv/C and Rv/δ cells, as indicated. The transfected cells were harvested and subjected to immunoblotting with the following antibodies: EGFR and GFP. GFP antibody is used to detect C-terminus of PS-1 expression (middle panel) and PS-1 high molecular weight complex (bottom panel). (**B**) δ-Catenin did not affect EGFR degradation induced by caspase-3. Caspase-3 plasmid was transfected into Rv/C and Rv/δ cells. The cells were harvested and subjected to immunoblotting with the following antibodies: EGFR, caspase-3 and β-actin. Caspase-3 antibody was able to detect both exogenous and endogenous caspase-3. Although exogenous caspase-3 was weak, it dramatically decreased EGFR expression. (**C**) δ-Catenin did not enhance EGFR protein expression under hypoxic condition. Rv/C and Rv/δ cells were treated with 200 μM CoCl_2_ for 24 h. The cells were harvested and subjected to immunoblotting with the following antibodies: EGFR, δ-Catenin, GFP and β-actin. (**D**) δ-Catenin did not dramatically change the effect of Fbw-7 on EGFR expression. Fbw-7-myc plasmid was transfected into both Rv/C and Rv/δ cells. The transfected cells were harvested and subjected to immunoblotting with the following antibodies: EGFR, myc and β-actin.

EGFR has been reported to be negatively regulated by caspase-3 and presenilin-1. In order to understand how δ-catenin enhanced the stability of EGFR, we investigated whether δ-catenin protected EGFR from degradation through caspase-3 and presenilin-1. We overexpressed caspase-3 or presenilin-1 in Rv/C and Rv/δ cells to determine their effects on EGFR protein expression. All of them expectedly induced a decrease in EGFR expression, which was consistent with previous reports. However, there was no significant difference in decreased rates of EGFR levels between the 2 cell lines (Fig. 7A,B). These results indicated that δ-catenin may not protect EGFR from degradation mediated by caspase-3 and presenilin-1 in CWR22Rv-1 cells.

It has been reported that hypoxic condition and Fbw-7 increase EGFR protein expression^5^,^6^. Therefore, we tested if δ-catenin protects EGFR under hypoxic condition or through Fbw-7. We firstly compared protein expression of EGFR in both Rv/C and Rv/δ cell lines either under normoxic or hypoxic condition. However, there was no significant difference between 2 cell lines in terms of changed EGFR expression induced by hypoxic condition (Fig. 7C). Next, we investigated whether δ-catenin increases EGFR expression through Fbw-7. As it shown in Fig. 7D, the effect of exogenous Fbw-7 on EGFR was similar in Rv/δ and Rv/C cells, indicating that δ-catenin may not mediate EGFR stability through Fbw-7 in CWR22Rv-1 cells.

### δ-Catenin enhanced EGFR/p-Erk1/2 signaling

**Figure 8:**
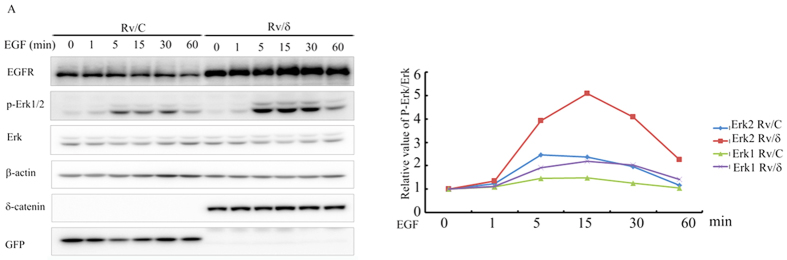
δ-Catenin enhanced the EGFR/Erk1/2 signaling in CWR22Rv-1 cells. Rv/C and Rv/δ cells were cultured in serum free condition for 16 h followed by treatment of 100 nM EGF for different duration, as indicated. The cells were harvested and subjected to immunoblotting with the following antibodies: EGFR, p-Erk1/2, Erk1 (detect both Erk1 and Erk2), β-actin, δ-catenin and GFP. These proteins were all from the same lysate. EGFR and β-actin were from the same gel; GFP and δ-catenin were from the same gel; Erk1/2 and p-Erk1/2 were from the same gel. The experiments were performed 5 times. The density of p-Erk1/2 bands were normalized by the density of total Erk1/2 bands. The relative values were shown in the right panel.

The protective role of δ-catenin on EGFR and the effect on EGFR mediated signaling was investigated by comparing the p-Erk1/2 expression between Rv/C and Rv/δ cells. One min post-treatment with EGF, p-Erk1/2 expression was similar in both cell lines. However, p-Erk1/2 expression was significantly higher in Rv/δ cells than that in Rv/C cells, at 5, 15, 30, and 60 min post-treatment. This result indicated that δ-catenin enhanced the EGFR/Erk1/2 signaling pathway (Fig. 8A).

## Discussion

We reported the interesting interaction between EGFR and δ-catenin and its potential effects on each of the proteins. We demonstrated that, on one hand, in the presence of EGF, EGFR phosphorylated δ-catenin in a Src independent manner, whereas on the other hand, δ-catenin enhanced protein stability of EGFR and strengthened the EGFR/Erk1/2 signaling pathway.

It has been shown that EGFR interacted with the E-cadherin-catenin complex through β-catenin^23^,^24^. Here, we reported that EGFR co-localized and interacted with δ-catenin in prostate cancer cells, adding a brand new perspective to the interaction of EGFR to the E-cadherin complex. Intriguingly, the binding of EGFR to δ-catenin was reduced in the presence of EGF. There are 3 potential mechanisms underlying the reduction in the interaction. Firstly, dimerization or endocytosis of EGFR may decrease its affinity to δ-catenin. In the presence of EGF, the binding of ligands initiates dimerization of EGFR, which in turn, leads to its endocytosis. Thus, either the conformational change of EGFR or endocytosis of EGFR upon stimulation with EGF could reduce its interaction to δ-catenin. Secondly, tyrosine phosphorylation of δ-catenin may induce changes in its conformation, which may lower its affinity for EGFR. Thirdly, tyrosine phosphorylation of δ-catenin may change its localization within cellular compartments; if phosphorylated-δ-catenin does not co-localize with EGFR in the same cellular compartment, the binding would decrease. Our fractionation study data suggested that EGF treatment did not significantly affect localization of δ-catenin. Therefore, we were able to rule out the third possibility. Moreover, our immunoprecipitation data showed that EGF treatment failed to change the binding affinity of δ-catenin to E-cadherin, indicating that it was unlikely that dramatic change in δ-catenin conformation occurred in the presence of EGF. Additionally, in the immunostaining experiments, while EGFR was significantly endocytosed in the presence of EGF, localization of δ-catenin rarely changed. Therefore, it was highly likely that EGF induced EGFR dimerization and endocytosis which in turn, reduced the interaction of EGFR to δ-catenin. Additionally, although the exact binding region on each protein is not clear, it appeared that the δ-catenin- EGFR was non-competitive with δ-catenin-E-cadherin, since EGF treatment changed the binding of δ-catenin to EGFR but did not change its binding to E-cadherin.

Previously, we have demonstrated that Src family kinases could mainly induce tyrosine phosphorylation of δ-catenin on its residues on C-terminus^22^. Interestingly, in this study, we observed that δ-catenin could be phosphorylated by EGF through EGFR, in a Src independent manner. Although the events of tyrosine phosphorylation triggered by EGF are generally *via* EGFR-Src to substrate proteins, it was not surprising to observe that EGF phosphorylated δ-catenin in a Src independent manner because of the following 2 reasons. Firstly, δ-catenin mainly binds to the juxta-membrane area of E-cadherin, which is very close to the cell membrane. On the other hand, as a transmembrane protein, EGFR has intrinsic kinase domains within the cytosol, which provides a spatial possibility for EGFR to phosphorylate δ-catenin without recruiting Src. Our immunoprecipitation and immunostaining data supported this possibility by showing the interaction or co-localization of EGFR to δ-catenin. Secondly, it was reported that p120ctn could be phosphorylated by EGF in a Src independent manner^19^. Therefore, our observation on the phosphorylation of δ-catenin was consistent with what has been reported on the phosphorylation of p120ctn.

Interestingly, we observed that δ-catenin stabilized EGFR in CWR22Rv-1 cells, and made diverse attempts to determine the underlying mechanism. On one hand, we tested the effect of exogenous caspase-3 and presenilin-1 on EGFR, either in the absence, or presence of δ-catenin. On the other hand, we checked the effect of δ-catenin on these negative regulators of EGFR. Our data suggested that neither of these negative regulators was involved in stabilization of EGFR mediated by δ-catenin in CWR22Rv-1 cells. These findings indicate that there might be other important regulators for EGFR. It is likely that δ-catenin protected EGFR directly or via as yet unidentified regulators. Future studies are required to identify the potential regulators.

We surprisingly detected 2 EGFR bands from membrane fractions purified by biotin. Interestingly, the bottom band was time-dependently reduced in response to EGF treatment whereas the upper band was relatively insensitive to the treatment. Furthermore, the expression pattern of these 2 bands were opposite in Rv/C and Rv/δ cell lines. The EGFR antibody that we used is known to potentially cross-react with Her2. Therefore, it is possible that one of bands was Her2. However, we have ruled out this possibility since we were unable to detect Her2 specific expression in lysates from both Rv/C and Rv/δ cells (Fig. 5A, right panel). The antibody does not cross-react with Her3 or Her4. Hence the upper band was likely to be a phosphorylated form of EGFR. Therefore, it might be phosphorylated by other kinases or ligands since EGF treatment failed to increase the upper band. We also used phospho-EGFR (Y1068) antibody to check if the upper band is auto-phosphorylation band. However, the phospho-EGFR bands could be detected only under the treatment of EGF (Fig. 6A, last panel), indicating that the upper band is not phospho-EGFR (Y1068).

Furthermore, loss of p120ctn is reportedly associated with augmentation of EGFR signaling in invasive breast cancer^18^. However, in the present study, we demonstrated that, unlike p120ctn, overexpression of δ-catenin enhanced EGFR signaling in CWR22Rv-1 prostate cancer cells. δ-catenin and p120ctn belong to the same subfamily and have similarities in structure and function. For example, both proteins bind to the juxta-membrane region of E-cadherin^13^,^14^. However, they have different expression patterns in prostate cancer with high Gleason scores. Along with increased Gleason scores, while p120ctn protein expression was significantly decreased, expression of δ-catenin protein was increased^16^,^17^, suggestive of different functions in late stage prostate cancer. Moreover, δ-catenin overexpression has been reported to mediate a decrease in p120ctn levels in CWR22Rv-1 cells^25^. Therefore, our observation on the role of δ-catenin in enhancing EGFR signaling in prostate cancer cells was consistent with the reported role of p120ctn on EGFR in breast cancer.

We demonstrated the interaction of EGFR to δ-catenin and the effects of this interaction on each of the associated proteins in prostate cancer cells. Not only did our findings add a new perspective to the interaction of EGFR to the E-cadherin complex but they also provided novel insights into the roles of δ-catenin in prostate cancer cells. The correlation of upregulated δ-catenin and the activation of EGFR signaling in either human prostate tumor tissues or *in vivo* mouse models of prostate cancer, remains to be further investigated in future studies.

## Methods

### Plasmids

The constructs of δ-catenin-GFP, δ-catenin-RFP, GFP-Presenilin-1 (G-PS-1), C-terminus of G-PS-1 and N-terminus of G-PS-1 have been described previously^26^,^27^.The constructs of pSH1/M-Fv-Casp3E (Addgene ID: 15271) was provided by Addgene. Fbw-7-myc and EGFR-GFP plasmids were kindly provided by Professor Kwang Youl Lee from Chonnam National University.

### Antibodies and reagents

Antibodies for immunoblotting were from commercially available sources. anti-δ-catenin (#611537, BD Bioscience); anti-GFP (#G1544, Sigma); anti-p-Erk1/2 (#16982, Santa Cruz); anti-Erk1 (SC-94, Santa Cruz); anti-EGFR 1005 (#03, Santa Cruz); anti-EGFR (D20) (#31156, Santa Cruz) anti-E-cadherin (SC-7870, Santa Cruz); anti-py20 (SC-508, Santa Cruz) and anti-Myc-Tag (9B11) (#2276,Cell signaling).

Antibodies for immunofluorescence were also obtained commercially: anti-E-cadherin (#3195, Cell signaling), anti-EGFR 225 (MA5-12880, ThermoFisher Scientific).

Control siRNA and siRNA anti-δ-catenin were purchased from Sigma-Aldrich.

### Cell culture and transfection

Bosc23 cells were cultured in DMEM supplemented with 10% FBS and 1% penicillin/streptomycin at 37 °C with 5% CO_2_. Rv/δ and Rv/C cells derived from CWR22Rv-1, overexpressing δ-catenin and GFP respectively, were maintained in RPMI supplemented with 10% FBS, 1% penicillin/streptomycin and G418 (Sigma, St Louis, MO) 125 ug/ml at 37 °C with 5% CO_2_. Bosc23 cells were transfected using calcium phosphate, while other cell lines were transfected with Lipofectamine Plus reagent (Invitrogen, Carlsbad, CA) according to the manufacturer’s instructions.

### Immunoblotting and immunoprecipitation

Immunoblotting was performed as previously described^28^. Immunoprecipitation was performed as follows. Briefly, lysates were incubated with primary antibodies for 16 h at 4 °C and pulled out with protein G sepharose (GE healthcare, Uppsala, Sweden) for 3 h. The immune-complexes were washed thrice, denatured at 95 °C for 2 minutes with 15 μl of 2X sample buffer (0.1 M Tris-HCl, pH6.8, 0.2 M DTT, 4% SDS, 20% glycerol, 0.2% bromophenol blue, 1.43 M β-mercaptoethanol) and loaded on a SDS-gel followed by immunoblotting analysis.

### Biotinylation

Biotinylation was performed as previously described^29^. Briefly, Rv/δ and Rv/C cells were cultured on 100 mm dishes and were collected for further experimentation post-EGF treatment. Specifically, cells were gently washed twice using ice-cold reaction buffer (0.1 M phosphate, 0.15 M NaCl, pH 8.0). Next, cell surface proteins were labeled with biotin by incubating with 0.5 mM Sulfo-NHS-SS-Biotin (Pierce, Rockford, IL) at room temperature for 40 min. Cells were subsequently lysed in MLB lysis buffer followed by centrifugation at 13,200 rpm for 15 min at 4 °C. Sample protein concentration was measured and equal amounts of protein lysates were explored by pull-down with streptoavidin-agarose beads (Pierce, Rockford, IL). This process resulted in purification of membrane proteins. The purified proteins were next subjected to immunoblotting. The biotinylated plasma membrane associated EGFR was detected by immunoblotting with an EGFR antibody. E-cadherin was used as a loading control.

### Immunofluorescence staining and image acquisition

Bosc23 and CWR22Rv-1 cells transfected with δ-catenin-RFP and EGFR-GFP were grown on glass coverslips. After 24 h of transfection, the cells were fixed with 4% PFA in PBS and subjected to confocal analysis. Rv/δ cells grown on glass coverslips were fixed and subjected to immunofluorescence staining using the anti-E-cadherin antibody and Alexa Fluor 633 Goat anti-mouse lgG. Stained coverslips were subjected to confocal analysis. Post-EGF treatment for different time duration, Rv/δ cells grown on glass coverslips were fixed and subjected to immunofluorescence staining using anti-EGFR antibody and Alexa 555 Goat anti-rabbit lgG. Images were visualized and acquired using a TCS SP5 AOBS/Tandem microscope (Leica Microscope system GmBh) at Korea Basic Science Institute, Gwangju.

### Q-PCR analysis

Total RNA (1 μg) from each cell was converted to cDNA using a M-MLV reverse Transcriptase kit (Invitrogen, Carlsbad, CA, USA). Quantitative analysis of target and reference genes was performed in triplicate using SYBR green (Enzynomics, Seoul, Korea) on CFX (Bio-Rad, Hercules, CA, USA).

The primers used for the reaction were EGFR (forward) 5′-tggaaaacctgcagatcatc-3′ and (reverse) 5′-ttgctgagaaagtcactgct-3′; GAPDH (forward) 5′-atcaccatcttccaggagcga-3′ and (reverse) 5′-agttgtcatggatgaccttggc-3′. Results are obtained from five independent experiments.

### Flow cytometry

Rv/C and Rv/δ cells were plated at 60 mm dish (1 × 10^6^ cells/dish). After overnight incubation, 40 μ M of CHX was added to each plate for indicated time durations (0, 4, 8, 12 h). Cells were washed with cold PBS and incubated with 1^st^Ab in FACS buffer (2% FBS in PBS) for 1 h on ice. After 1^st^Ab attachment, cells were extensively washed with FACS buffer and further incubated with PE fluorochrome solution for 30 min in dark condition. When staining with fluorochrome is over, the cells were washed with FACS buffer for 2 times and resuspended in FACS buffer. The samples are transferred to FACS tubes and applied to FACSCalibur™ for detection of the EGFR expression on the surface of each cell. Experiments were done for three times with independent trials.

## Additional Information

**How to cite this article**: He, Y. *et al.* Interaction of EGFR to δ-catenin leads to δ-catenin phosphorylation and enhances EGFR signaling. *Sci. Rep.* **6**, 21207; doi: 10.1038/srep21207 (2016).

## Supplementary Material

Supplementary Figure 1-3
