# Supplementary material for: Interaction of EGFR to δ-catenin leads to δ-catenin phosphorylation and enhances EGFR signaling
Source: Sci Rep. 2016 Feb 17;6:21207. doi: 10.1038/srep21207 (PMC4756308; doi:10.1038/srep21207)
Supplement: Supplementary Figure 1-3 [file srep21207-s1.pdf]

Interaction of EGFR to δ-catenin leads to δ-catenin phosphorylation and enhances EGFR signaling

Yongfeng He[1], Taeyong Ryu[1], Nensi Shrestha[1], Tingting Yuan[1], Hangun Kim[2], Hridaya Shrestha[1], Young-Chang Cho[3], Young-Woo Seo[4], Woo Keun Song[5], Kwonseop Kim[1*]

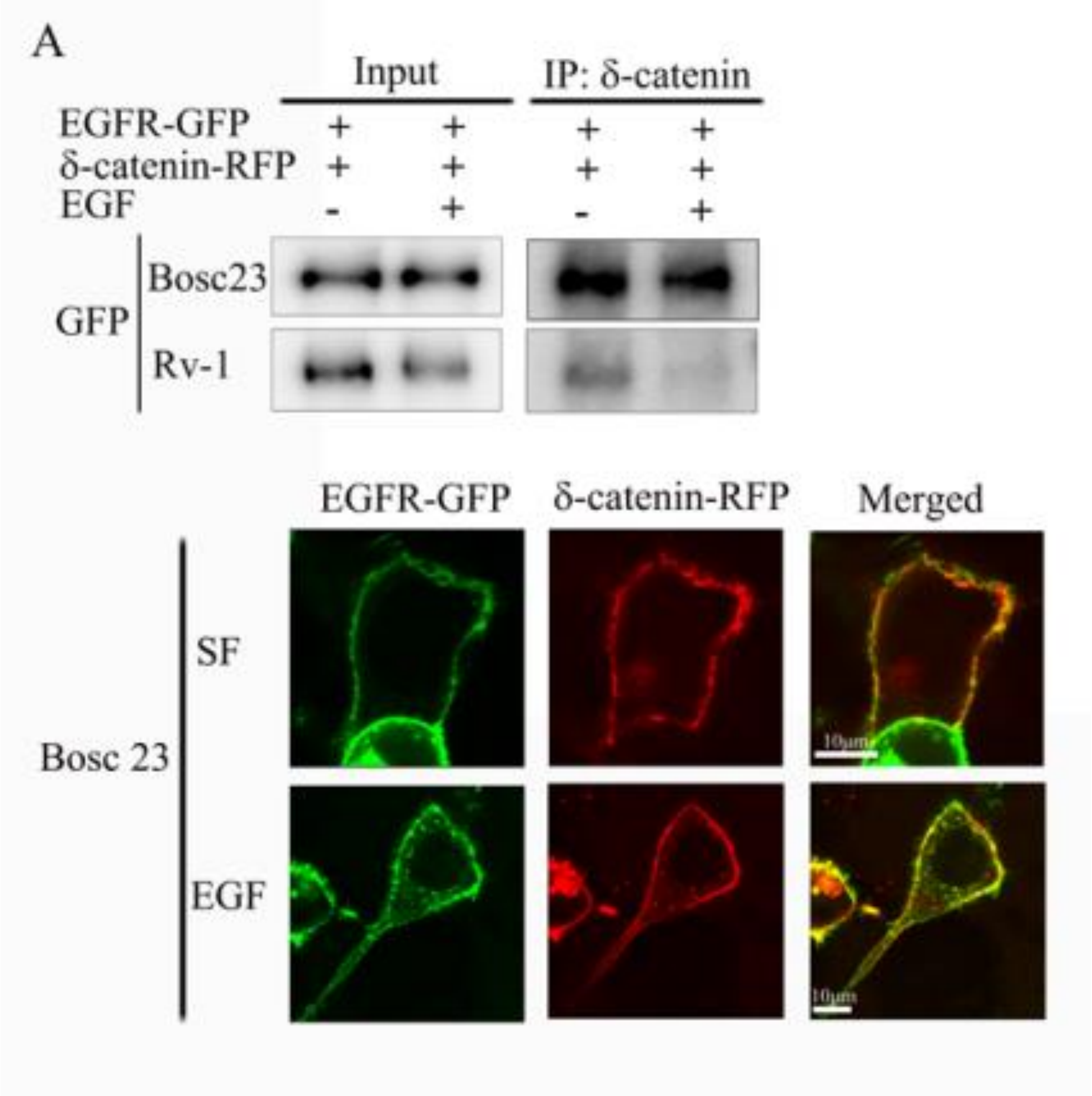

Figure S1. δ-Catenin interacted with EGFR in an EGF dependent manner in CWR22Rv-1 cells and Bosc23 cells. (A) Both CWR22Rv-1 cells and bosc23 cells were transfected with δ-catenin-RFP and EGFR-GFP. The cells were treated with EGF 100 nM for 30 minutes and harvested to perform immunoprecipitation with anti-δ-catenin antibody followed by immunoblotting with anti-GFP (EGFR) antibody. (B) Transfected Bosc23 cells (δ-catenin-RFP, EGFR-GFP) on coverslips were treated with EGF 100 nM for 5 minutes and then subjected to confocal microscopy analysis. There experiments were performed 3 times.

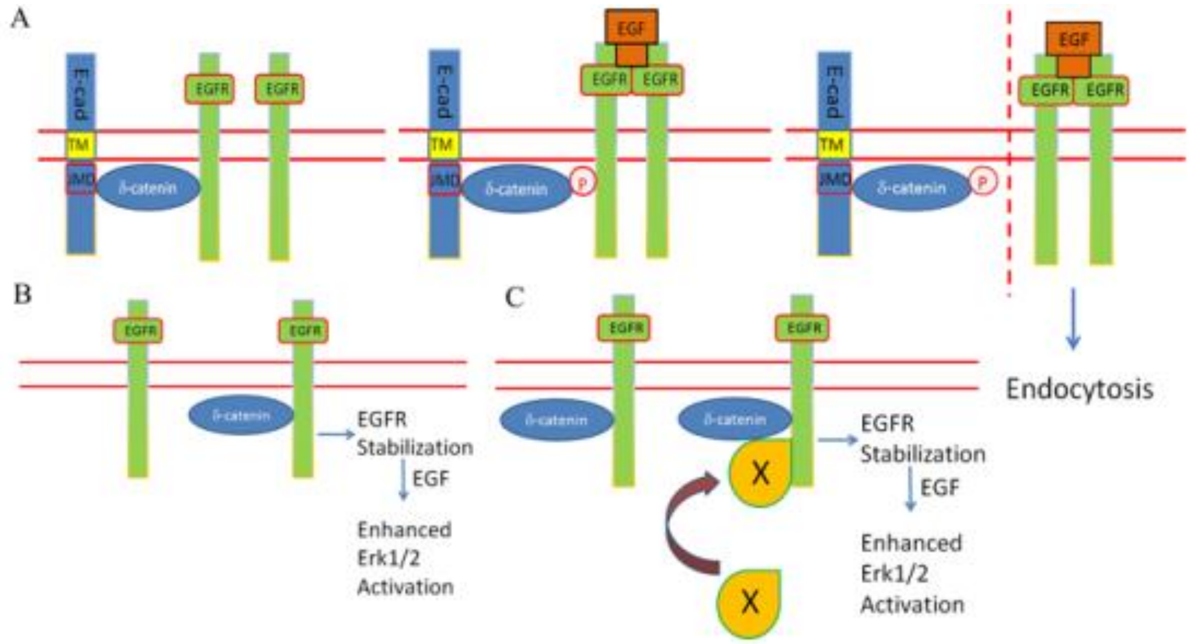

Figure S2. Proposed models illustrating the effect of EGF on interaction of δ-catenin and EGFR and the effect of δ-catenin on EGFR. (A) EGF decreased interaction of EGFR to δ-catenin through inducing endocytosis of EGFR. (B, C) δ-catenin enhanced EGFR/Erk1/2 signaling through stabilizing EGFR either directly or via other unknown factors.

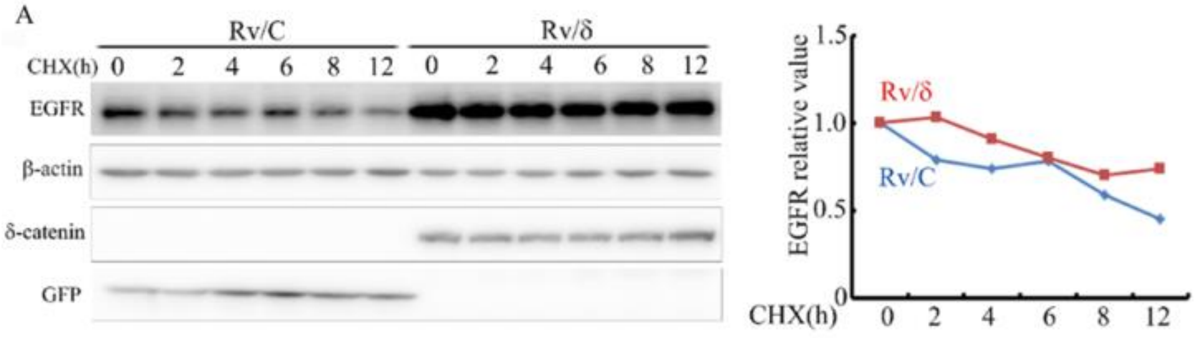

Figure S3. δ-Catenin enhanced EGFR protein stability. Both Rv/C and Rv/δ cells were treated with 40 μM cycloheximide (CHX) for different durations, as indicated. The cells were harvested after the treatment and subjected to immunoblotting with anti-EGFR, anti-δ-catenin, anti-GFP and anti-β-actin antibodies. The density of EGFR protein was normalized by β-actin. And the band densities observed from the lysates without CHX treatment were considered as value 1, although the EGFR band density in Rv/δ was much higher than that in Rv/C cells. Comparison of EGFR relative values was demonstrated in the right panel. The GFP and δ-catenin bands were from the same gel and the EGFR and actin bands were from the same gel.
